# A bright single-cell resolution live imaging reporter of Notch signaling in the mouse

**DOI:** 10.1186/1471-213X-13-15

**Published:** 2013-04-25

**Authors:** Sonja Nowotschin, Panagiotis Xenopoulos, Nadine Schrode, Anna-Katerina Hadjantonakis

**Affiliations:** 1 Developmental Biology Program, Sloan-Kettering Institute, New York, NY, USA

**Keywords:** Mouse embryo, Live imaging, Cell tracking, GFP, Venus, H2B-Venus, Reporter strain, CBF/RbpJ, Notch signaling

## Abstract

**Background:**

Live imaging provides an essential methodology for understanding complex and dynamic cell behaviors and their underlying molecular mechanisms. Genetically-encoded reporter expressing mouse strains are an important tool for use in live imaging experiments. Such reporter strains can be engineered by placing *cis*-regulatory elements of interest to direct the expression of desired reporter genes. If these *cis*-regulatory elements are downstream targets, and thus activated as a consequence of signaling pathway activation, such reporters can provide read-outs of the signaling status of a cell. The Notch signaling pathway is an evolutionary conserved pathway operating in multiple developmental processes as well as being the basis for several congenital diseases. The transcription factor CBF1 is a central evolutionarily conserved component of the Notch signaling pathway. It binds the active form of the Notch receptor (NICD) and subsequently binds to *cis*-regulatory regions (CBF1 binding sites) in the promoters of Notch responsive genes. In this way, CBF1 binding sites represent a good target for the design of a Notch signaling reporter.

**Results:**

To generate a single-cell resolution Notch signaling reporter, we used a CBF responsive element to direct the expression of a nuclear-localized fluorescent protein. To do this, we linked 4 copies of a consensus CBF1 binding site to the basal simian virus 40 (SV40) promoter, placed this cassette in front of a fluorescent protein fusion comprising human histone H2B linked to the yellow fluorescent protein (YFP) Venus, one of the brightest available YFPs. We used the *CBF:H2B-Venus* construct to generate both transgenic embryonic mouse stem (ES) cell lines and a strain of transgenic mice that would report Notch signaling activity.

**Conclusion:**

By using multiple CBF1 binding sites together with a subcellular-localized, genetically-encoded fluorescent protein, H2B-Venus, we have generated a transgenic strain of mice that faithfully recapitulates Notch signaling at single-cell resolution. This is the first mouse reporter strain in which individual cells transducing a Notch signal can be visualized. The improved resolution of this reporter makes it ideal for live imaging developmental processes regulated by the Notch signaling pathway as well as a short-term lineage tracer of Notch expressing cells due to the perdurance of the fluorescent reporter. Taken together, the *CBF:H2B-Venus* mouse strain is a unique tool to study and understand the morphogenetic events regulated by the Notch signaling pathway.

## Background

The Notch signaling pathway is one of the key signaling pathways employed during embryonic development, homeostasis and disease progression (reviewed in [1,2]). Though first discovered in *Drosophila*, the Notch pathway is evolutionary conserved across many metazoan species playing an important role in various processes including cell fate specification, differentiation, tissue patterning and homeostasis, as well as stem cell self-renewal (reviewed in [1][2]).

The core components of the Notch signaling pathway include the Notch transmembrane receptors, Notch1-4 in mammals, and their five canonical membrane-bound ligands of the Delta or Serrate/Jagged family, Jagged1-2, and Delta1, Delta3 and Delta4 in mammals. Notch proteins are single-pass transmembrane proteins consisting of an extracellular domain (NECD), encompassing 29–36 tandem epidermal growth factor like repeats, a shorter membrane-spanning region and an intracellular domain (NICD) containing a transactivation domain (reviewed in [1]).

Cell-cell contact and the interaction of the NECD with one of the ligands expressed by a neighboring cell leads to a cascade of proteolytic events involving an S2 cleavage of the Notch receptor, and a cleavage at the S3 site by γ-secretase. This series of proteolytic cleavages results in the release of the NICD from the membrane. Thus, upon signaling the NICD translocates to the nucleus where it binds the transcription factor CBF1 (also referred to as RBPJκ, CSL or Su(H)). The binding of NICD to CBF1 replaces co-factors of the Groucho family that function to repress target genes and in doing so allows the recruitment of the co-activator mastermind-like (MAML) promoting the formation of a CBF1-NICD-MAML complex that leads to transcriptional activation of target genes including members of the Hes and Hey families of bHLH transcription factors [1].

Transgenic reporters providing *in vivo* read-outs of Notch signaling activity have been useful for investigating the complex functions of Notch signaling across species. To date, several transgenic strains of mice have been established to monitor Notch signaling activity, each employing slightly different construct designs, but all based on the use of Notch-responsive elements driving the expression of a readily detectable genetically-encoded reporter. Of these, a transgenic mouse line, named NAS (for Notch Activity Sensor), used the *TP1* minimal promoter (comprising 12 multimerized CBF binding sites), which is transactivated in a CBF/RBPJκ-dependent manner by the NICD and in doing so functions as a read-out of Notch activity. The NAS reporter comprised a modified version of the *lacZ* gene coding for nuclear ß-galactosidase under the control of *TP1*. Faithful expression regulated by Notch signaling was demonstrated by loss of reporter activity in *CBF* mutants [3]. Another transgenic mouse line, *CBF:EGFP*, used EGFP as a reporter gene driven by the basal simian virus (SV40) promoter and a CBF1-responsive element containing four CBF1 binding sites. The *CBF:EGFP* strain has been used to investigate Notch signaling in a variety of contexts, including neural stem cells and intermediate neural progenitors during mouse neural development. Faithful responsiveness of the *CBF:EGFP* to Notch signaling activity was demonstrated using an shRNA mediated knock-down of CBF1 and the γ–secretase antagonist F18 [4].

A recent report developed a construct based on the promoter of the Notch target gene *Hes5-1*, coupled with a destabilized nuclear-localized Venus fluorescent protein and the 3’ untranslated region of *Hes5-1*. Using transient transgenesis by electroporation, this Notch signaling reporter was used in chick embryos to investigate endogenous Notch activity during neurogenesis. Highlighting the importance of single-cell resolution reporters to understand cell fate and lineage decisions, this reporter revealed distinct Notch signaling dynamics in different cell division modes in neural progenitors within the neuroepithelium [5].

Another report presented an alternative strategy to the use of transcriptional reporters as read-outs of Notch signaling activity that could also be used for monitoring Notch activation in real-time in living cells. In this newly described reporter Notch activity was recorded in a cell culture system using a luciferase complementation-based reporter that could directly monitor interactions between a specific NICD and CBF/RBPJκ, thereby allowing the non-invasive detection of protein-protein interactions in any subcellular compartment [6].

Since the available Notch reporter mouse strains either lack sensitivity or do not provide single-cell resolution read-outs, we sought to generate an improved reporter that would provide single-cell resolution reporter activity, as a key in understanding cell dynamics and behaviors, and greater sensitivity by incorporating a nuclear-localized bright fluorescent reporter, which could be readily detected, live imaged and quantified. To do so, we designed a construct that combined the signaling read-out efficiency of the multimerized *CBF1* responsive elements [4] with an improved nuclear-localized fusion to a bright fluorescent protein. Fluorescent proteins fused to histones are bound to chromatin even during cell division, therefore allowing the tracking of single cells and their progeny, as well as providing information on cell division and cell death [7,8]. We generated an H2B-Venus fusion protein, comprising human histone H2B fused to the bright yellow fluorescent protein Venus, and placed it under the control of four CBF1 responsive elements and the simian virus 40 (SV40) minimal promoter in a configuration resembling the previously reported *CBF:EGFP* Notch signaling reporter [4]. We used this construct to generate *CBF:H2B-Venus* transgenic mouse ES cells and an equivalent strain of mice. In this way the *CBF:H2B-Venus* transgene was designed to function as a sensitive single-cell resolution reporter of Notch signaling, and would be comparable to the *TCF/Lef:H2B-GFP* single-cell resolution reporter we previously generated as a read-out of WNT/β-catenin signaling [9].

We recovered several founder transgenic lines, which recapitulated the spatio-temporal localization of Notch signaling activity during mouse embryonic development, as well as in adults showing among others expression in the headfolds, neural tissue of the early embryo, the vasculature, the kidney, and brain. One was analyzed in detail and is presented here. Overall, expression of *CBF:H2B-Venus* recapitulated the previously reported *CBF:EGFP* localization including the expression in the ventricular zone (VZ) and sub-ventricular zone (SVZ) regions of the cortex of the brain [4]. We confirmed Notch signaling specificity of the reporter, both in cells and in embryos, through Notch signaling stimulation achieved by misexpression of NICD leading to reporter activation.

The *CBF:H2B-Venus* reporter strain produced bright single-cell resolution reporter activity. Notch responsiveness of the *CBF:H2B-Venus* reporter was demonstrated through reporter activation by transfection of an NICD expression construct in mouse embryo fibroblast (MEF) cells derived from transgenic embryos, and electroporation into the visceral endoderm of early post-implantation embryos. Due to its improved sensitivity, the *CBF:H2B-Venus* reporter revealed additional sites of Notch signaling activity, that have previously not been described, including expression in the epiblast (EPI) of the peri-implantation mouse embryo. Collectively, these data demonstrate that we have generated a transgenic mouse strain that faithfully reports Notch signaling activity and serves as a quantitative, non-invasive single-cell resolution reporter of Notch signaling. The *CBF:H2B-Venus* reporter currently represents an improved tool for imaging the *in vivo* processes triggered by canonical Notch signaling.

## Results and discussion

### Generation of *CBF:H2B-Venus* reporter construct

**Figure 1:**
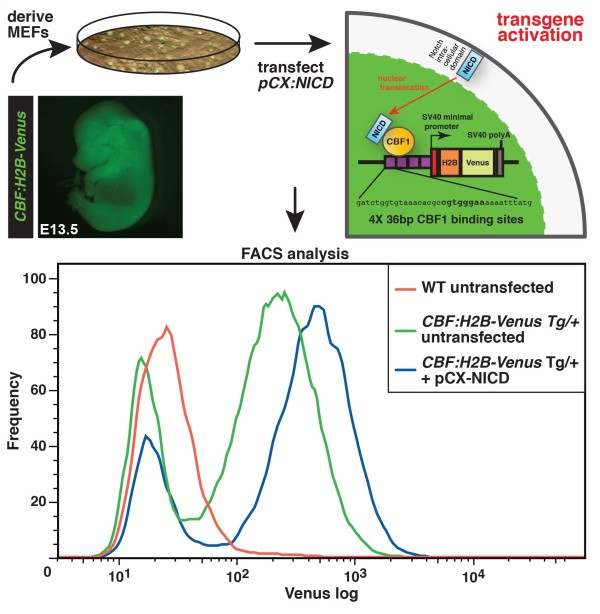
***CBF:H2B-Venus *construct design and validation in MEF cells. ***CBF:H2B-Venus *MEFs are derived from E13.5 transgenic embryos and transfected with a *pCX:NICD *construct. Ectopic expression of NICD activates the canonical Notch signaling pathway and in turn leads to transgene expression, which can be assessed using flow cytometry. FACS analysis shows transgenic *CBF:H2B-Venus *MEFs comprise a Venus negative and Venus positive population and display an increase in fluorescence upon ectopic NICD expression.

Several *cis*-regulatory elements necessary for Notch signaling activated transcription have previously been tested in characterized Notch reporter lines [3,4], however, none of these lines provides a single-cell resolution read-out of Notch signaling activity. To overcome this issue, we used a previously characterized CBF1 responsive element to drive H2B-Venus reporter expression. The CBF1-responsive element (CBFRE) contains four CBF1-binding sites upstream of the basal simian virus-SV40-promoter (Figure 1) and has been used in previous studies reporting endogenous Notch signaling during mouse brain development [4]. The *CBFRE* and the *SV40* minimal promoter were cloned in front of an H2B-Venus reporter cassette to generate the final construct, *CBF:H2B-Venus*. We used this construct to generate transgenic *CBF:H2B-Venus* ES cells by electroporation. *CBF:H2B-Venus* transgenic mice were generated by pronuclear injection of the plasmid construct according to standard protocols. Three founder mice were identified and screened for bright and faithful expression of the reporter. The characterization of one of these is presented in this report.

### Reporter activation upon Notch signaling stimulation

To test for Notch responsiveness of the *CBF:H2B-Venus* construct, and in doing so validate the reporter as *a bona fide* Notch signaling reporter, we derived MEF cells from E13.5 hemizygous transgenic embryos and transfected them with an NICD expression construct (*pCX:NICD*) [4] to activate reporter expression. Subsequent FACS analysis revealed an increase in fluorescence upon transfection of *pCX:NICD* into *CBF:H2B-Venus*^*Tg/+*^ MEF cells demonstrating the Notch responsiveness of the reporter construct (Figure 1).

**Figure 2:**
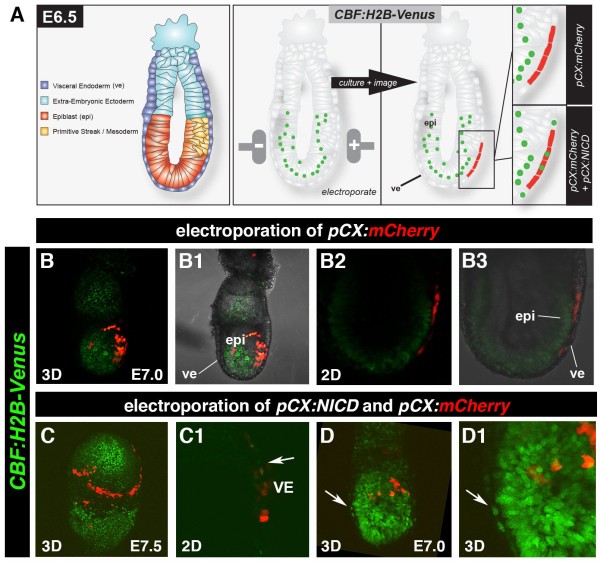
**Validation of the *CBF:H2B-Venus *reporter construct in embryos. **Schematic of electroporation of a *pCX:NICD* and *pCX:mCherry *construct into the ve of an E6.5 embryo (**A**). Electroporation of *pCX:mCherry *into a *CBF:H2B-Venus *transgenic embryo and subsequent culture shows expression of mCherry but no reporter activation in the ve (**B**). Electroporation of *pCX:NICD *and *pCX:mCherry *into a *CBF:H2B-Venus *transgenic embryo and subsequent culture shows expression of mCherry as well as reporter activation in the ve (**C**, and **D**, white arrowheads). Of note, the ve does not normally express the nuclear-localized Venus reporter, though cytoplasmic fluorescence which is background fluorescence likely due to fixation of samples is sometimes observed (see Figures 4B and D). mCherry is not localized in the epiblast (epi), these 3D projections depict reporter expression on the embryos surface in the ve layer (see panels B3 and B1). Cells appear yellow due to strong Venus expression in epiblast (epi), this bleeds through from the underlying epiblast layer in 3D projections. By contrast, 2D data (see panels B2 and B3) reveal that there are no green (Venus-positive) cells in ve which comprises only mCherry-positive cells.

To validate the reporter *in vivo*, we electroporated the *pCX:NICD* plasmid into the superficial visceral endoderm (VE) layer of E6.5 *CBF:H2B-Venus*^*Tg/+*^ embryos (Figure 2). The VE does not normally express the H2B-Venus reporter in wild-type embryos. An *pCX:mCherry* construct, providing constitutive expression of the mCherry cytoplasmic red fluorescent protein was either electroporated alone (as a control for the electroporation, Figure 2B-B3) or together with the *pCX:NICD* plasmid (Figure 2C-D1). Electroporated and control non-electroporated embryos were then subject to *ex utero* culture [10]. Cells receiving the *pCX:mCherry* would be readily visualized due to cytoplasmic mCherry red fluorescence (Figure 2A), while cells receiving the *pCX:NICD* would activate the *CBF:H2B-Venus* reporter and be visualized by their nuclear-localized Venus fluorescence, and cells receiving both *pCX:NICD* and *CBF:H2B-Venus* would exhibit a red fluorescent cytoplasm and yellow fluorescent nuclei. Indeed, electroporation of *pCX:mCherry* alone did not activate the H2B-Venus reporter (Figure 2B-B3). By contrast, electroporation of *pCX:NICD* did activate reporter expression in cells of the VE (Figure 2C, C1, D, D1, white arrows in C1, D and D1) thereby demonstrating the Notch responsiveness of the *CBF:H2B-Venus* reporter.

### Generation of *CBF:H2B-Venus* transgenic ES cells

**Figure 3:**
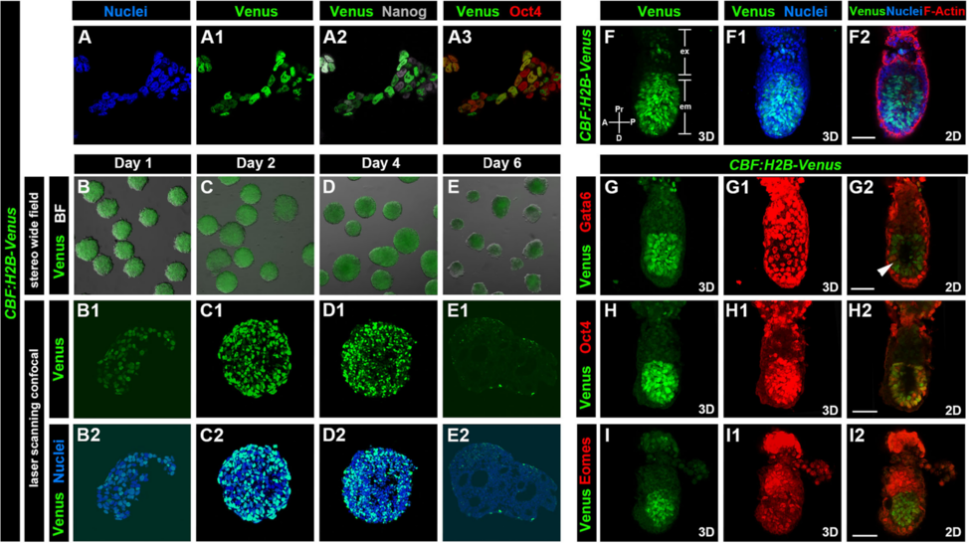
**Expression of *CBF:H2B-Venus *in ES cells and pre-streak stages. **Immunostaining of the *CBF:H2B-Venus* ES cells (**A**-**A3**) for pluripotency-associated factors Nanog (grey) (**A2**) and Oct4 (red) (**A3**). Nuclei were stained with Hoechst (blue) (**A**). Embryoid body formation from *CBF:H2B-Venus *ES cells. Fluorescence was assessed after 1, 2, 4 and 6 days of EB culture (**B**-**E**). Expression of *CBF:H2B-Venus* in the epiblast at E6.0 (**F**) and E5.5 (**G**-**I**). Expression of Gata6 (**G1**, **G2**), Oct4 (**H1**, **H2**), and Eomes (**I1**, **I2**) in *CBF:H2B-Venus *embryos. At E6.0 *CBF:H2B-Venus* expression is detected in the epiblast in a salt and pepper distribution as well as in the extra-embryonic ectoderm (**F**, **F1**, **F2**). At E5.5 *CBF:H2B-Venus* expression is restricted to the epiblast (**G**, **H **and **I**). H2B-Venus positive cells express also Oct4 (**H2**). Note, Gata6 expression in the epiblast (white arrowhead, **G2**). Embryo has been counterstained with Hoechst (**F1**, **F2**) and Phalloidin (**F2**) to label nuclei and F-Actin. A, anterior; D, distal; em, embryonic; ex, extra-embryonic; P, posterior; Pr, proximal. Scale bars: 50 μm.

Since previous studies have reported that mouse ES cells express Notch receptors and ligands [11], we investigated whether the *CBF:H2B-Venus* transgene was expressed in pluripotent mouse embryonic stem (ES) cells. The *CBF:H2B-Venus* transgene was introduced into R1 ES cells [12] and stable transgenic lines were selected (Figure 3A-A3). Laser scanning confocal imaging of transgenic ES cells revealed that the *CBF:H2B-Venus* reporter was nuclear-localized in individual cells and overlapped with a Hoechst nuclear stain (Figure 3A-A1)). The reporter was present in a subpopulation of ES cells expressing the pluripotency-associated factors Nanog (Figure 3A2) and Oct4 (Figure 3A3).

Since it had previously been reported that Notch signaling is involved in regulating the timing of the emergence of the different germ layers in ES cell-derived embryoid bodies (EBs), we used EB formation to test the differentiation potential of the *CBF:H2B-Venus* transgenic ES cells and determine whether reporter localization might correlate with the timing of germ layer formation (Figure 3B-E) [13]. Our results revealed that Notch signaling could initially be detected in EBs cultured until Day 4 (Figure 3B-D). By day 6 however, fluorescence was downregulated in concordance with progressive differentiation. At this stage tube-like or cystic structures could be detected within EBs where Notch signaling had been downregulated (Figure 3E) [14].

Collectively, these data indicate that the *CBF:H2B-Venus* transgene is a sensitive single-cell resolution Notch signaling reporter that reveals Notch signaling activity in mouse ES cell cultures.

### The *CBF:H2B-Venus* reporter reveals novel sites of Notch signaling within the early post-implantation epiblast

The robust expression of the *CBF:H2B-Venus* transgenic ES cells prompted us to investigate whether the reporter is also active in the pluripotent epiblast lineage of the pre-implantation embryo in *CBF:H2B-Venus* transgenic embryos. We failed to detect any fluorescent signal within the inner cell mass of E3.5-E4.5 transgenic blastocysts (data not shown), suggesting that either the *CBF:H2B-Venus* reporter was not sufficiently sensitive for direct visualization at these stages, or that Notch signaling is not active in the epiblast lineage at pre-implantation stages.

To further characterize the localization of the *CBF:H2B-Venus* reporter and thus the activation of Notch signaling, we analyzed its distribution in early post-implantation stages (Figure 3F-I). After implantation into the maternal uterus around embryonic day (E) 4.5, the three blastocyst cell lineages (epiblast, primitive endoderm and trophoblast) are expanded and the axes of the embryo (proximal-distal/P-D and anterior-posterior/A-P) are specified at early post-implantation (~E5.5-E6.0). The visceral endoderm (VE) encapsulates the pluripotent epiblast distally, and extra-embryonic ectoderm proximally, and close apposition of these adjacent tissues facilitates cross talk between them, resulting in the formation of the distal visceral endoderm (DVE). Subsequently, translocation of the DVE population to generate the anterior visceral endoderm (AVE) leads to the establishment of the A-P axis (reviewed in [15]).

At the earliest post-implantation stages that we could recover (~E5.0-5.25), reporter expression was detected in the epiblast of the *CBF:H2B-Venus* embryos (Figure 3F, F1, F2, G, G2, H, H2, I and I2). Expression in the epiblast persisted until the onset of gastrulation (Figure 3F and data not shown). In addition, for a very brief window of time, at around ~ E6.0, reporter expression was detected in the extra-embryonic ectoderm (Figure 3F). This extra-embryonic expression was downregulated by ~ E6.5 (data not shown). Of note, reporter expression in the EPI at early post-implantation stages looks like a salt-and-pepper distribution (Figure 3F1), begging the question whether this distribution represents different cell populations or whether these could be fluctuations in Notch signaling. Future studies will address this question. Epiblast-specific location was confirmed by co-localization with the epiblast marker, Oct4 (Figure 3H). Localization of the pan-VE marker Gata6 (Figure 3G), and pan-emVE marker Eomes in *CBF:H2B-Venus* embryos revealed that the reporter expression was excluded from the VE (Figure 3G, I). The epiblast-specific site of Notch reporter expression has not been previously reported and may presage the future requirement for Notch signaling and subsequently reporter expression in neural tissue.

### *The CBF:H2B-Venus* reporter marks the neural plate and cardiogenic region at gastrulation

During gastrulation, which in the mouse takes place between E6.5 and E8.0, three germ layers, ectoderm, mesoderm and definitive endoderm, are formed from the pluripotent epiblast lineage. Mesoderm and definitive endoderm arise when epiblast cells undergo an epithelial-to-mesenchymal transition (EMT), and in doing so lose their epithelial character, ingress through the primitive streak (PS), which forms at the posterior end of the embryo, and migrate away as the so-called wings of mesoderm [15,16].

**Figure 4:**
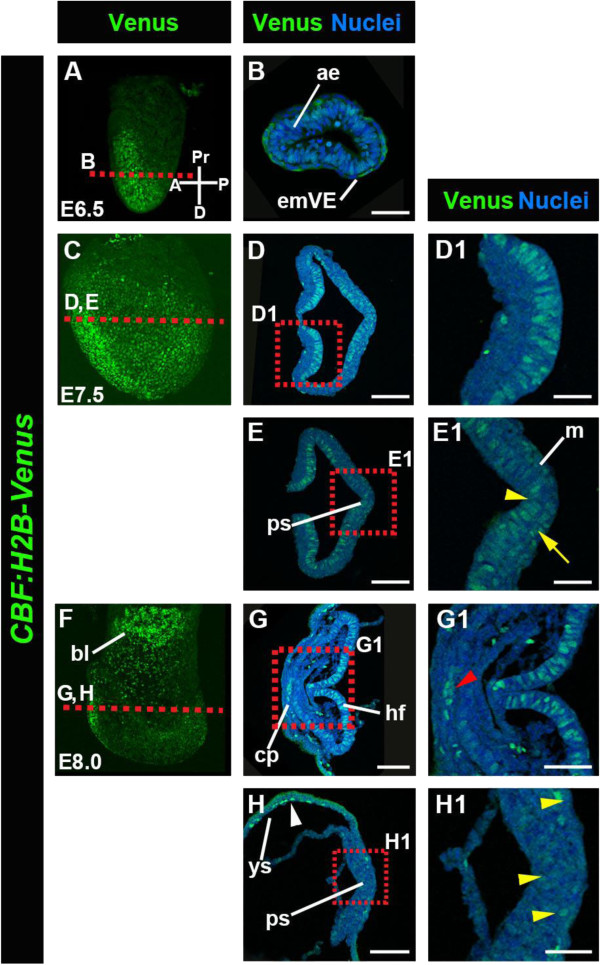
**Expression of *CBF:H2B-Venus *in mouse embryos at E6.5-E8.0. **Expression of *CBF:H2B-Venus *in E6.5 embryos (**A**-**B**) showing strong expression in the anterior epiblast (ae, **B**, **B1**). Whole mount view of an E6.5 embryo (**A**). Transverse section through an E6.5 embryo (**B**). Expression of *CBF:H2BVenus *in E7.5 embryos (**C**-**E**). Lateral whole mount view of an E7.5 embryo (**C**). Transverse section through the anterior and posterior of an E7.5 (**D**, **D1**, **E **and **E1**) showing expression in the neural plate (**D**, **D1**) and in the primitive streak (ps) region in the epiblast (yellow arrowhead in **E1**), as well as in the nascent mesoderm (yellow arrow in **E1**). Expression of *CBF:H2B-Venus* in E8.0 embryos (**F**-**H**). Lateral whole mount view of an E8.0 embryo (**F**). Transverse section through the anterior and posterior section of an E8.0 showing expression in the headfolds (**G** and **G1**), cardiogenic plate (cp, red arrowhead, **G1**) in the mesodermal layer of the yolk sac (ys, **H**) and cells in the primitive streak region (yellow arrowheads, **H2**). All sections were counterstained with Hoechst to label nuclei. A, anterior; bl, blood islands; D, distal; emVE, embryonic visceral endoderm; m, mesoderm, P, posterior; Pr, proximal. Scale bars: 100 μm (**D**, **E**, **G**, **G1 **and **H**); 50 μm (**B**, **E1 **and **H1**); 30 μm (**D1**).

**Figure 5:**
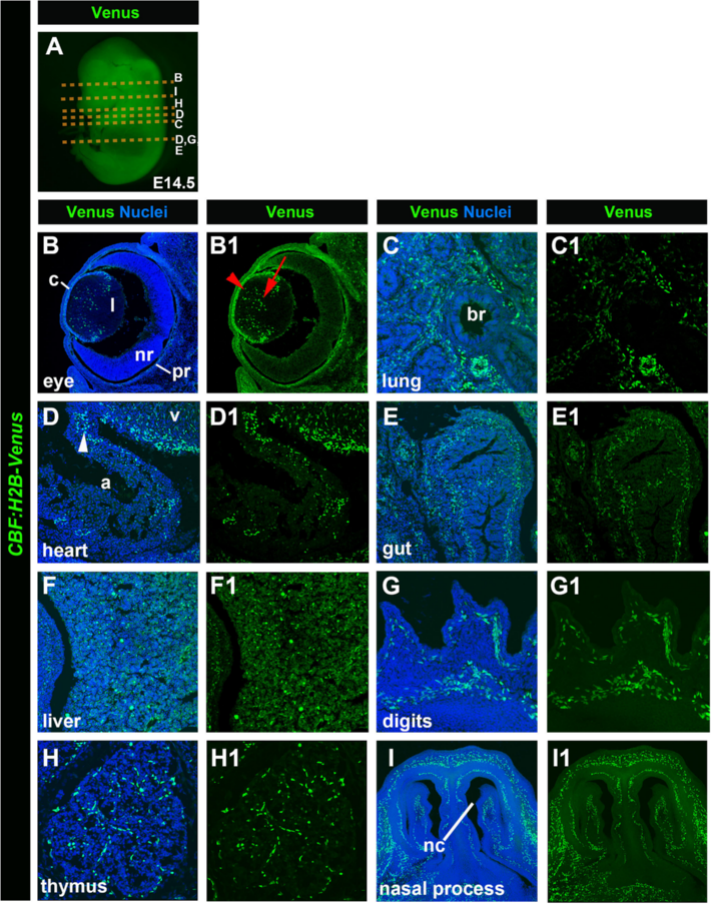
**Expression of *CBF:H2B-Venus *at fetal stages (~E14.5). **Stereo widefield image of a lateral view of an E14.5 embryo expressing *CBF:H2B-Venus *(**A**). Transverse sections of E14.5 embryo showing *CBF:H2B-Venus* reporter expression (**B1**, **C1**, **D1**, **E1**, **F1**, **G1**, **H1**, **I1**) and *CBF:H2B-Venus* expression and Hoechst to label nuclei, respectively (**B**, **C**, **D**, **E**, **F**, **G**, **H**, **I**). *CBF:H2B-Venus *expression at E14.5 in the lens (**l**) and the pigmented part of retina (pr) (**B**,**B1**). Red arrowhead points to expression in cuboidal epithelium in anterior part of the lens. Red arrow points to Notch expression in cells of equatorial region of the lens. Expression of *CBF:H2B-Venus *in the interstitial mesenchymal cells of the lung (**C**, **C1**), in cells of the forming valves of the heart (**D**, **D1**), the gut (**E**, **E1**), the liver (**F**, **F1**) as well as in the vasculature of the digits (**G**, **G1**), cells of the thymus (**H**, **H1**) and nasal process (**I**, **I1**). a, atrium; br, bronchus; c, cornea; nc, nasal cavity; nr, neural part of retina, v, ventricle.

To evaluate *CBF:H2B-Venus* Notch reporter expression at gastrulation stages, we examined embryos between E6.5 and E8.0 (Figure 4). At E6.5 strong reporter expression in *CBF:H2B-Venus* embryos was found in the anterior epiblast, in the prospective headfold region in contrast to lower level of fluorescence in the posterior part (Figure 4A-B). At E7.5 the reporter continued to be strongly expressed in cells of the forming headfolds (Figure 4C-D), and in cells within the PS region. In the latter it could be detected in both, epiblast (yellow arrowhead, Figure 4E1) and nascent mesoderm cells (yellow arrow in Figure 4E1). Reporter expression in cells in the headfolds persisted until later stages, ~E8.0 (Figure 4F-G). At E8.0 single-positive cells with variable fluorescent intensities could also be detected in the PS region of the embryo (yellow arrowheads in Figure 4H1). In addition, at this stage reporter expression could also be noted in the cardiogenic plate (Figure 4G and G1, red arrowhead), in agreement with a previous report in the chick [17]. Notably, the role of Notch signaling in early cardiac cells in the mouse is still elusive, whereas its role in later stages of heart development, especially in valve formation, has been well documented [18] (see also Notch reporter expression in the valve in Figure 5D, D1). In the latter report, Notch1 activity was analyzed using an antibody against the N1ICD and could detect expression in the nascent mesoderm, however in contrast to the *CBF:H2B-Venus* reporter, no expression could be detected in the neuroectoderm [18]. A possible explanation for this discrepancy could be that the *CBF:H2B-Venus* reporter is a transcriptional not translational reporter of Notch signaling activity.

In addition, robust reporter expression was detected in the blood islands (Figure 4F), a cell population of maturing erythrocytes surrounded by developing endothelial cells that will give rise to the mesoderm layer of the yolk sac [19], another site of persistent specific and strong Notch reporter expression (Figure 4H white arrowhead).

Surprisingly, we failed to observe expression of the *CBF:H2B-Venus* reporter in the presomitic mesoderm (PSM), a tissue of known and dynamic Notch activity [20]. However, several previously characterized reporters have also failed to detect Notch activity at this site of Notch activity.

### *CBF:H2B-Venus* reporter expression at mid-gestation and to later fetal stages

**Figure 6:**
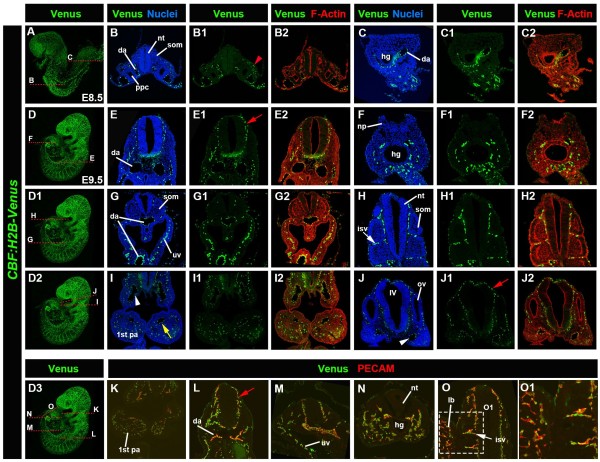
**Expression of *CBF:H2B-Venus *reporter at E8.5-E9.5 with specific localization in endothelial cells.** Expression of *CBF:H2B-Venus *in E8.5 embryos (**A**-**C**). Lateral whole mount view of an E8.5 embryo (**A**). Transverse sections through the anterior and posterior regions of E8.5 embryo depicting reporter expression in dorsal aorta (da) (**B**-**C**) as well as intermediate mesoderm (red arrowhead, **B1**). Expression of *CBF:H2B-Venus *at E9.5 (**D**-**J**). Lateral views of same E9.5 embryo to highlight the plane of section (**D**, **D1**, **D2**, **D3**). Transverse sections through anterior and posterior regions of E9.5 embryo depicting reporter expression in da (**E**, **E1**, **E2**, **G**, **G1**, **G2**), head vessels (**E**, red arrow in **E1**, **E2**, **J**, red arrow in **J1 **and **J2**), umbilical veins (**G**, **G1**, **G2**), intersomitic vessels (isv, white arrow in **H**, **H1 **and **H2**), pharyngeal arch arteries (yellow arrow in **I**, **I1 **and **I2**) and mesoderm of the pharyngeal arches (pa, **I**, **I1**, **I2 **and **I3**). Expression of *CBF:H2B-Venus *counterstained with Hoechst to label nuclei in E8.5 embryo (**B**, **C**), in E9.5 embryo (**E**, **F**, **G**, **H**, **I**, **J**). Expression of *CBF:H2B-Venus *in E8.5 (**B1**, **C1**) and E9.5 (**E1**, **F1**, **G1**, **H1**, **I1**, **J1**) embryos. Expression of *CBF:H2B-Venus* counterstained with phalloidin to label F-Actin in E8.5 (**B2**, **C2**) and E9.5 (**E2**, **F2**, **G2**, **H2**, **I2**, **J2**). Co-staining with PECAM (**K**-**O** and **O1**), a marker for endothelial cells in E9.5 reveals localization of *CBF:H2B-Venus* reporter expression to endothelial cells in the pharyngeal arches (**K**), dorsal aorta (**L**), head vessels (**L**), umbilical veins (**M**), tail bud (**N**), intersomitic vessels (**O**, white arrow) and limb bud (**O**). IV, fourth ventricle; hg, hindgut; lb, limb bud; np, neural plate; nt, neural tube; ov, otic vesicle; ppc, pericardio-peritoneal canal; som, somites; uv, umbilical veins.

By midgestation *CBF:H2B-Venus* reporter expression was predominantly localized in the vasculature. The *CBF:H2B-Venus* reporter was observed in endothelial cells of the dorsal aorta at E8.5 and at E9.5, as well as in the umbilical vessels (Figure 6A-C, E and L). Expression was also detected in the intersomitic vessels (Figure 6H and O), the pharyngeal arch arteries (Figure 6I and K), vessels of the head surrounding the neural tube and the otic vesicle (Figure 6E, J, L and M), as well as in the developing vasculature of the limb bud (Figure 6O).

We determined that this observed site of reporter expression was in endothelial cells of the vasculature, by analyzing the localization of PECAM (CD31) a marker of endothelial cells in *CBF:H2B-Venus* embryos and noting the co-localization of the PECAM antibody and Venus (Figure 6K-O). Note, the nuclear expression of the reporter and membrane expression of PECAM in the same cells (Figure 6O1).

Localization of *CBF:H2B-Venus* expression at later fetal stages (E14.5, Figure 5) revealed reporter expression in a variety of tissues. In the lens, H2B-Venus could be detected in cells of the cuboidal epithelium that comprises the anterior lens (red arrowhead), as well as in cells in the equatorial region (red arrow), the region where anterior capsular epithelial cells become integrated into the lens-proper and form the lens fibers. In the retina of the eye, strong expression could be found in the retinal pigmented epithelium, RPE (Figure 5B and B1). Reporter expression was also detected in mesenchymal cells of the lung (Figure 5C and C1), in cells that will form the valves of the heart (Figure 5D and D1), in cells of the gut (Figure 5E and E1) and liver (Figure 5F and F1), in the vasculature of the digits (Figure 5G and G1), as well as in cells of thymus (Figure 5H and H1), and in cells surrounding the nasal process likely being vascular cells (Figure 5I and I1).

### *CBF:H2B-Venus* reporter expression in post-natal tissues

**Figure 7:**
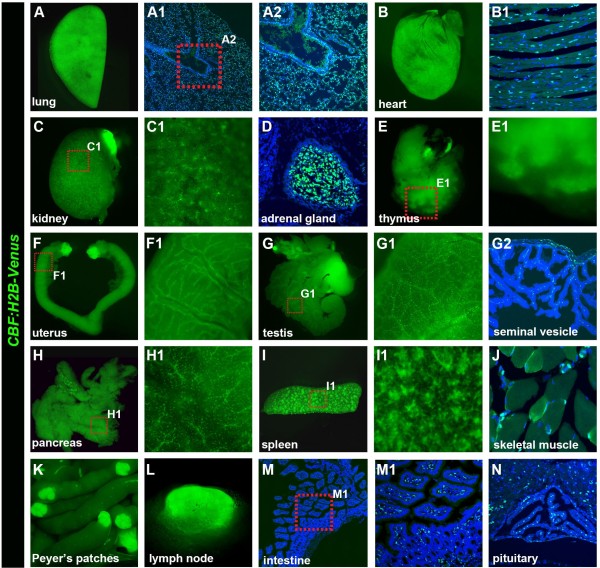
**Expression of *CBF:H2B-Venus *in adult mouse organs. **Widefield fluorescent images of *CBF:H2B-Venus* expression in the adult lung (**A**), heart (**B**), kidney (**C**, **C1**), thymus (**E**, **E1**), uterus (**F**, **F1**), testis (**G**, **G1**), pancreas (**H**, **H1**), spleen (**I**, **I’**), Peyer’s patches (**K**), lymph node (**L**) and eyeball (**O**). Laser confocal images of sections through the heart muscle (**B1**), adrenal gland (**D**), seminal vesicle (**G2**), skeletal muscle (**J**), intestine (**M**, **M1**) and pituitary (**N**). Sections are counterstained with Hoechst to label nuclei.

To examine the expression and localization of this novel Notch signaling reporter at postnatal tissues, we dissected organs of three-week-old animals and analyzed transgene expression (Figure 7). Strong reporter expression was found in the interstitial mesenchymal cells of the lung (Figure 7A, A1 and A2), as well as in the kidney (Figure 7 C and C1) and the adrenal gland (Figure 7D). Robust but restricted reporter expression, mainly to the vasculature, was observed in several tissues including the uterus, testis and pancreas (Figure 7F, F1, G, G1, G2, H and H1). Reporter expression was also detected in cells of the cardiac and skeletal muscle (Figure 7B, B1 and J). Recent studies have revealed that Notch signaling is required for the establishment and maintenance of muscle satellite cells, confirmation of whether Venus-positive cells are satellite cells, is outside the scope of the present study and will be addressed in the future (reviewed in [2][21]). Notch signaling has been implicated in T-cell lineage commitment and maturation in the thymus, as well as in T-cell activation and differentiation (reviewed in [22]). Fluorescence indicative of Notch signaling activity was detected in several parts of the immune system of the animal, such as in cells of the thymus, the spleen, Peyer’s patches within the intestine, and the lymph nodes (Figure 7E, E1, I, I1, K and L). Additional sites of *CBF:H2B-Venus* reporter expression that can be noted were the intestine (Figure 7M and M1) and the pituitary (Figure 7N).

### *CBF:H2B-Venus reporter* expression in the kidney

To validate the *CBF:H2B-Venus* reporter as an improved tool for visually dissecting morphogenetic processes, we chose to look at reporter expression in the kidney in detail. The importance of the evolutionary conserved Notch signaling pathway is not just illustrated by the fact that it is indispensable for kidney development across phyla [23], but it is also involved in the pathophysiology of congenital diseases, such as the Alagille syndrome [24,25], glomerular diseases, in tissue repair after acute renal injuries [26][27], as well as in the prevention of renal epithelial cancers [28].

**Figure 8:**
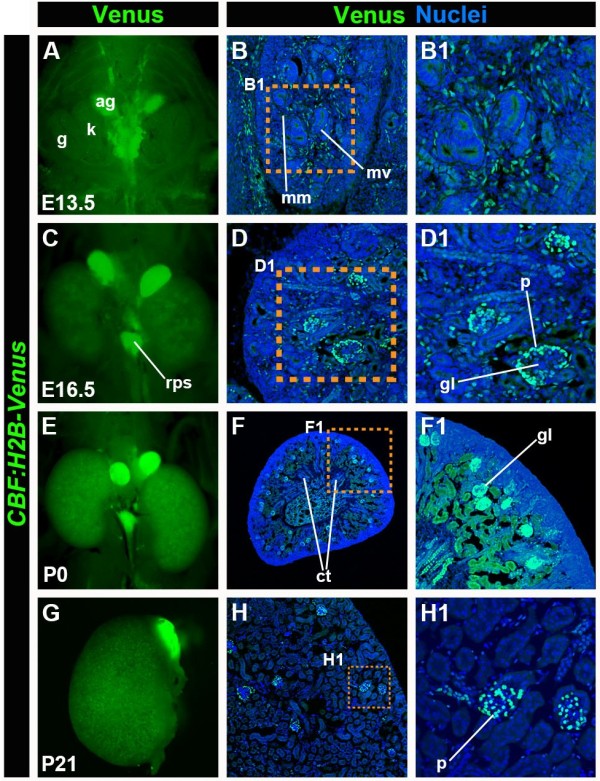
***CBF:H2B-Venus *expression during kidney development. **Fluorescent stereo-widefield images of whole mount kidneys at stages E13.5 (**A**), E16.5 (**C**), P0 (**E**) and P21 (**G**) showing *CBF:H2B-Venus *expression. Laser confocal images of transverse sections through the kidney of stages E13.5 (**B**, **B1**), E16.5 (**D**, **D1**), P0 (**F**, **F1**) and P21 (**H**, **H1**) expressing *CBF:H2B-Venus*. Sections are stained with Hoechst to label nuclei. ag, adrenal gland; ct, collecting tubules; g, gonad; gl, glomerulus, k, kidney; mm, mesenchymal tissue of medullary region; mv, metanephric vesicle, p, podocytes. Note, strong expression in the retro-peritoneal lymph sac (rps).

The mammalian kidney develops from the intermediate mesoderm in three distinct phases, successively forming the pro-, meso- and metanephros. Whereas the metanephros will give rise to the adult kidney, the pro- and mesonephros are vestigial embryonic structures that will eventually degenerate. The pronephros begins to develop at around E10.5 giving rise to the ureteric bud (UB), which will invade the metanephric mesenchyme surrounding it. Bifurcation of the UB and subsequent branching and elongation will form the collecting duct system. Reciprocal signaling between the metanephric mesenchyme and the UB is important to give rise to the progenitor cells of glomerular and epithelial cells. Since Notch signaling has been shown to play a key role in these processes, we analyzed reporter expression in the developing kidney during metanephros development at E13.5 and E16.5 (Figure 8A, B, B1 and C, D, D1) as well as neo- (P0) (Figure 8E, F, F1) and postnatal (P21) stages (Figure 8G, H and H1). At E13.5 we could detect reporter expression in the metanephric mesenchyme (Figure 8B, B1). By E16.5 through postnatal stages, *H2B-Venus* expression was observed in the podocytes (Figure 8C, C1). Notch-2 has previously been reported to be required for the differentiation of proximal nephron structures, such as podocytes and proximal convoluted tubules [29]. At neonatal (P0) stages, *H2B-Venus* expression was detected in the collecting duct system as well as in the podocytes (Figure 8F-F1). In three-week old (P21) animals reporter expression was exclusively observed in the podocytes (Figure 8H-H1).

### *CBF:H2B-Venus* reporter expression in the brain

**Figure 9:**
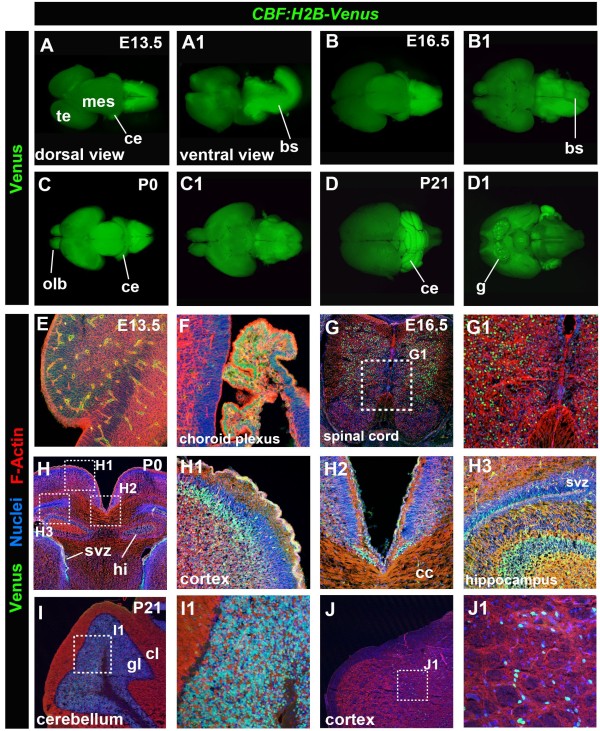
***CBF:H2B-Venus *expression during brain development. **Epifluorescent widefield images of E13.5 (**A**, **A1**), E16.5 (**B**, **B1**), P0 (**C**, **C1**) and P21 (**D**, **D1**) depicting *CBF:H2B-Venus* expression in the dorsal (**A**, **B**, **C**, and **D**) and ventral (**A1**, **B1**, **C1 **and **D1**) part of the brain. Laser confocal images of sections through the brain of stages E13.5 (**E**, **F**), E16.5 (**G**, **G1**), P0 (**H**, **H1**, **H2**, **H3**) and P21 (**I**, **I1**, **J** and **J1**) expressing *CBF:H2B-Venus *stained with Hoechst and Phalloidin to label nuclei and F-Actin, respectively. Lateral section through the telencephalon of an E13.5 brain (**E**). Lateral section through the choroid plexus at E13.5 (**F**). Transverse section through the brain stem area at E16.5 (**G**, **G1**). Frontal sections through a P0 brain (**H**, **H1**, **H2**, **H3**). Transverse sections through a P21 brain (**I**, **I1**, **J **and **J2**). bs, brain stem; cc, Corpus Callosum: ce, cerebellum; cl, cortical layer; g, ganglium; gl, granular layer; hi, hippocampus; mes, mesencephalon; olb, olfactory bulb; svz, subventricular zone; te telencephalon.

Notch signaling has been intensively studied and shown to be indispensable for various aspects of neural development. Notch signaling has been proposed as a master regulator of neural stem cells, as well as serving crucial functions in differentiated cells in the adult brain. We therefore analyzed the localization of the *CBF:H2B-Venus* reporter in the developing brain (Figure 9). Even though our analysis is not comprehensive, it identifies areas of Notch signaling in the brain that are in agreement with previously published domains of Notch expression [4].

**Figure 10:**
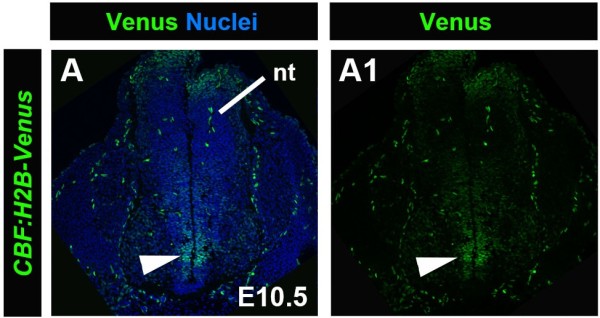
***CBF:H2B-Venus *expression in the V2 zone of the neural tube. **Laser confocal images of transverse sections through the neural tube region of a E10.5 embryo showing expression of *CBF:H2B-Venus *in the ventricular zone 2 (V2) (white arrowhead, **A **and **A1**). *CBF:H2B-Venus *expression counterstained with Hoechst to label nuclei (**A**). *CBF:H2B-Venus *expression only (**A1**). nt, neural tube.

In general overview, from embryonic through adult stages reporter expression was observed in several parts of the brain including the telencephalon, mesencephalon, the olfactory bulbs, ganglia, as well as the cerebellum and the brain stem (Figure 9A-D). The latter two exhibited very strong *CBF:H2B-Venus* reporter expression. A more detailed histological analysis of brain sections revealed expression at E13.5 in neurons in the cortex (Figure 9E) and in the choroid plexus (Figure 9F). Reporter expression was noted in the spinal cord from E13.5 to adulthood, and is depicted at E16.5 (Figure 9G, G1). We also detected expression in the hippocampus and the subventricular zone (SVZ), as well as in the cortex. A particular group of cells in the cortex at the border of the two hemispheres overlying the corpus callosum exhibited very strong expression (Figure 9H2). In the cerebellum, shown at P21, robust reporter expression was detected in the granular layer, whereas only a few sparse cells in the cortical layer were positive for the reporter (Figure 9I, I1). At this stage reporter expressing cells, likely neurons, were also detected in the cortex (Figure 9J, J1). During early embryogenesis, at E10.5, we also observed *CBF:H2B-Venus* reporter expression in the ventricular zone 2 (V2) of the neural tube (Figure 10, white arrowhead) Expression of Notch and its ligands in V2 has been previously described [30,31].

## Conclusions

We have generated a novel single-cell resolution transgenic strain of mice that serves as a transcriptional read-out of Notch signaling. The reporter comprises multimerized CBF1 DNA binding sites that drive the expression of a subcellular-localized, genetically-encoded fluorescent fusion protein, H2B-Venus. We have characterized the expression of the *CBF:H2B-Venus* reporter in detail in mouse ES cells, post-implantation embryos and adult tissues. Specificity of this putative Notch signaling reporter was demonstrated by its activation upon transfection with an NICD expression construct in transgenic MEF cells grown in culture and *in vivo* in embryos, as well as by documenting regions of reporter expression that correspond to those previously reported as sites of Notch signaling activity. Collectively, these data suggest that *CBF:H2B-Venus* reporter expression acts as a faithful read-out of Notch signaling.

In addition to previously reported sites of expression, the sensitivity of the reporter identified new sites of Notch signaling in embryos, in particular within the epiblast of the early post-implantation embryo. These novel sites of expression are supported by previous observations of Notch signaling function made in mouse embryonic stem (ES) cells.

Even though the reporter described in this report provides a read-out of the transcription resulting from pathway activation, and is not a read-out of the localization of one of the core components of the Notch signaling pathway, we cannot exclude some perdurance of the fluorescent protein. As such, the complete domain of reporter expression, that we have characterized, may encompass cells actively signaling as well as those that have recently been actively signaling but currently not signaling. Future efforts will be aimed at generating more sensitive reporters and will include the development of dual reporters that combine short-term expression, using a destabilized fluorescent protein with a more perdurant fluorescent protein as has recently been described in zebrafish [32].

Even so, the *CBF:H2B-Venus* reporter is, at present, the most sensitive reporter of Notch signaling, and due to the use of the human histone H2B fusion, it allows the live visualization of individual cells in cohorts. It is presently the only reporter facilitating the analysis of cellular dynamics, including tracking of single cells in Notch responsive populations. Live imaging has become an essential part of understanding developmental processes; therefore this reporter should be a valuable tool to understand cellular events in development and disease regulated by canonical Notch signaling.

## Methods

### Generation of Notch reporter constructs and transgenic animals

To generate the *CBF:H2B-Venus* construct, a CBF responsive element (CBFRE) including 4 binding sites for CBF1 together with basal simian virus 40 (SV40) promoter was amplified from the *CBFRE:EGFP* plasmid (purchased from Addgene, plasmid #17705), which has been described previously [4]. The amplified CBF responsive element was then subcloned into the *Ase*I/*Nhe*I sites of *pCMV:H2B-Venus.* Recombinant clones were confirmed with restriction digest analysis as well as sequencing. Transgenic mice were generated by pronuclear injection following standard protocols. Animals were genotyped by PCR using either of two alternative strategies. Primers used for the PCR amplification of the *CBF responsive element* (*CBFRE*, 350bp) were CBF *Ase*I Fw: GCTGATTAATCGAGATCTGGTGTAAACAC and SV40 *Nhe*I Rev: GCGAGCTAGCCAGCTTTTTGCAAAAGCCTAG, or alternatively, a 475bp fragment from the Venus cassette was amplified using primers IMR872 Fw: AAGTTCATCTGCACCACCG and IMR873 Rev: TGCTCAGGTAGTGGTTGTCG. Several transgenic founder lines exhibiting comparable expression were identified. One transgenic founder line (*CBF:H2B-Venus* #5) was analyzed in detail and presented in this report. Subsequent generations exhibited Mendelian transgene inheritance, stable transgene activity and comparable levels of reporter expression. The transgene could be homozygozed without incurring any phenotype. Animals were maintained in accordance with National Institute of Health (NIH) guidelines for the care and use of laboratory animals and under the approval of the Memorial Sloan Kettering Cancer Center (MSKCC) Institutional Animal Care and Use Committee (IACUC).

### Generation of transgenic ES cells

The *CBF:H2B-Venus* construct was tested in R1 ES cells [33]. R1 ES cells were cultured in standard conditions [34]. Transgenic R1 ES cells were generated by nucleofecting a *Fsp*I linearized *CBF:H2B-Venus* construct followed by selection of clones under Neomycin selection according to standard protocols.

### Transfection of MEFs

MEFs were derived from E13.5 transgenic embryos and passaged 3 times in Dulbecco's Modified Eagle Medium (DMEM; Gibco) containing 15% serum (Gibco) and 50 mg/mL each of penicillin/streptomycin (Gibco). They were subsequently seeded onto a 12-well plate (BD Falcon) and transfected with 2 μg *pCX:NICD*[4] (purchased from Addgene, plasmid #26891) using 10 μL Lipofectamine LTX (Invitrogen) and 2 μL Plus Reagent according to the manufacturer's protocol. Cells were then cultured for 36 h and analyzed using flow cytometry.

### Generation of embryoid bodies (EBs)

ES cells were passaged off MEFs, as previously described [35], then plated as hanging drops at a concentration of 15,000 cells/mL (300 cells per hanging drop) in Iscove's Modified Dulbecco Medium (IMDM; Gibco) containing 20% serum (Gibco), 50 mg/mL each of penicillin/streptomycin (Gibco), 200 mg/mL apo-transferrin (Sigma), 5% protein-free hybridoma medium (PFHMII; Gibco), 0.5 mM 1-thioglycerol (Sigma), and 0.5 mM ascorbic acid (Sigma). Ebs were harvested after 1, 2, 4 and 6 days of culture and imaged.

### FACS analysis

MEF cells were harvested 36hrs after transfection by trypsinization with 0.025% trypsin/EDTA (Invitrogen) and analyzed by flow cytometry using a FACS Fortessa flow cytometer (BD Biosciences). The live single cell population was selected for further analysis on the basis of Forward Scatter (FSC) and Side Scatter (SSC) characteristics as well as with Propidium Iodide (PI) staining to evaluate cell viability. Histogram plots of the data were generated using FlowJo (Tree Star, Inc).

### Embryo collection and culture

Early post-implantation mouse embryos were dissected in DMEM/F12 (1:1) containing 5% newborn calf serum and fixed in 4% paraformaldehyde. E13.5, E 16.5, P1 were dissected in PBS and fixed in 4% paraformaldehyde.

### Electroporation and culture of postimplantation embryos

E6.5 embryos were dissected and kept in culture medium (50% DMEM/F12, 50% rat serum). Then embryos were transferred into a drop of PBS. Highly concentrated plasmid DNA of *pCX:NICD* (purchased from Addgene, plasmid #26891) and/or *pCX:mCherry* was added and incubated for 5min. Subsequently, embryos were transferred into Tyrode’s ringer saline for electroporation. Embryos were pulsed using a ECM830 electroporator (BTX, Harvard Instruments). Settings were as follows: Voltage: 0017V, Length: 050ms; Pulse: 5, Interval 1s). Embryos were then cultured for 8hrs or over night in roller culture and then imaged. *pCX:mCherry* was generated by PCR of *mCherry* from *pRSET:mCherry*[36] using the 5’ oligo CCGGAATTCCGGATGGTGAGCAAGGGCGAGGAGGATAAC and the 3’oligo CCGGAATTCCGGTTACTTGTACAGCTCGTCCATGCCGCCGGT, both containing EcoRI restriction sites. The resulting PCR product cloned into pCAGGS [37] using the EcoRI restriction enzyme.

### Embryo sectioning

For cryosections, samples were equilibrated in 10% sucrose in PBS, followed by 30% sucrose in PBS overnight and OCT (Tissue-Tek) overnight, and then snap-frozen in OCT. Sections were cut with a Leica cryostat at 12 μm and counterstained with Hoechst 33342 (Invitrogen) to label nuclei. Sections were mounted in Fluoromount-G (Southern Biotech) and imaged through glass coverslips.

### Immunostaining

ES cells cultured on coverslips were immunostained as previously described [38]. Primary antibodies used were: NANOG (1:200, Cosmo Bio) and OCT4 (1:200, Santa Cruz). Embryos were permeabilized in 0.5% Triton X-100 in PBS for 20 minutes, washed in 0.1% Triton X-100 in PBS (PBT) and blocked in horse serum in PBT for 1 hr at 4C. Primary antibodies used were: anti-Eomes (1:500, Abcam), anti-Oct4 (1:200, Santa Cruz) anti-Gata6 (1:100, RD Systems) and anti-PECAM (CD31) (1:250, BD Pharmingen). Secondary Alexa Fluor conjugated antibodies were used at a dilution of 1/1000. Nuclei were visualized using Hoechst (Invitrogen). Phalloidin was used to label F-Actin.

### Image acquisition

Widefield and epifluorescence images were acquired using a Zeiss Axiocam MRc CCD camera coupled to a Leica M165FC dissecting scope. Laser scanning confocal data were acquired using a Zeiss LSM510META or a Zeiss LSM700 using a Fluar 5×/NA0.25, a PlanApo 10×/NA 0.45 and PlanApo 20×/NA0.75 objective. Fluorophores were excited using a 405 nm diode laser (Hoechst), 488 nm Argon laser (GFP) or 543 nm HeNe laser (Alexa 546) on the LSM 510 META, and 405 nm (Hoechst), 488 nm (GFP), 555 nm (Alexa 546) solid-state lasers on the LSM 700.

Embryos and ES cells were imaged in whole mount in MatTek dishes (Ashland). Confocal images were acquired as *z*-stacks of *xy* images taken at 2 μm *z*-intervals (20×) 10 μm z-intervals (10×) and 40 μm *z*–intervals (5×). Raw data were processed using the Zen 2010 software (Zeiss) and assembled in Adobe Photoshop CS4 (Adobe).

## Endnotes

The CBF:H2B-Venus transgenic strain described in this report has been accepted for inclusion in, and will be made available from, The Jackson Laboratory Mouse Repository as JAX Stock No. 020942 STOCK Tg(Cp-HIST1H2BB/Venus)#Hadj/J.

## Competing interests

The authors declare that they have no competing interests.

## Authors’ contributions

SN-designed and carried out experiments analyzing the *CBF:H2B-Venus* reporter in embryoid bodies, embryos and adult mice, and wrote the manuscript. PX-designed and carried out experiments to construct the *CBF:H2B-Venus* reporter, generated and analyzed *CBF:H2B-Venus* transgenic transgenic ES cells. NS generated and analyzed *CBF:H2B-Venus* transgenic MEFs. AKH-conceived, designed, funded and supervised the project. All authors critically read and revised the manuscript, and approved the final version.
